# Metabolic Tumour Volume as a Predictor of Survival for Sinonasal Tract Squamous Cell Carcinoma

**DOI:** 10.3390/diagnostics12010146

**Published:** 2022-01-07

**Authors:** Hidenori Suzuki, Tsuneo Tamaki, Takeshi Kodaira, Masami Nishio, Daisuke Nishikawa, Shintaro Beppu, Hoshino Terada, Michi Sawabe, Nobuhiro Hanai

**Affiliations:** 1 Department of Head and Neck Surgery, Aichi Cancer Center Hospital, Nagoya 464-8681, Japan; dsknishi@aichi-cc.jp (D.N.); ncu.beppin3@gmail.com (S.B.); hoshinoterada@aichi-cc.jp (H.T.); m.sawabe@aichi-cc.jp (M.S.); hanai@aichi-cc.jp (N.H.); 2 Department of Radiology, Nagoya Radiological Diagnosis Foundation, Nagoya 464-8681, Japan; ttamaki@kaikou.or.jp (T.T.); m-nishio@nagoya-pet.or.jp (M.N.); 3 Department of Radiation Oncology, Aichi Cancer Center Hospital, Nagoya 464-8681, Japan; 109103@aichi-cc.jp

**Keywords:** metabolic tumour volume, sinonasal tract, squamous cell carcinoma, predictor, cancer-specific survival

## Abstract

Background: High uptake of F18-fluorodeoxyglucose parameters for glucose metabolism is related to shorter survival in sinonasal tract cancer with various histological classifications. We investigated whether F18-fluorodeoxyglucose uptake parameters are associated with survival outcomes for patients with only squamous cell carcinoma (SCC) in the sinonasal tract that are treated either with surgery or nonsurgery. Methods: We retrospectively observed F18-fluorodeoxyglucose uptake parameters on positron emission tomography with computed tomography for the primary tumour of SCC in 39 patients. Log-rank test or a Cox regression model with 95% confidence interval (95%CI) and hazard ratio (HR) were used for monovariable or multivariable analysis, respectively. We determined cut-off values of the F18-fluorodeoxyglucose uptake parameters using the lowest *p* value for monovariable sinonasal tract cancer-specific survival analysis. Results: Monovariable analysis showed that patients with metabolic tumour volume (MTV) ≥ 21.8 had a shorter cancer-specific, disease-free and local recurrence-free survival than those with MTV < 21.8. After adjusting for age, gender, clinical stage and treatment group in the multivariable analysis, MTV (≥21.8/<21.8) was related to shorter cancer-specific (HR: 3.69, 95%CI: 1.17–12.0), disease-free (HR: 3.38, 95%CI: 1.19–9.71) and local recurrence-free (HR: 5.42, 95%CI: 1.59–20.3) survivals. Conclusions: MTV as advances in diagnostics of sinonasal tract SCC is a predictor.

## 1. Introduction

Squamous cell carcinoma (SCC) in the sinonasal tract is definitively treated by surgery or radiotherapy with or without chemotherapy [1]. In the initial staging of multiple organ cancer, volumetric fluorine-18-fluorodeoxyglucose uptake (F18-FDG uptake) on positron emission tomography with computed tomography (PET/CT) is acknowledged as a survival parameter [2,3,4,5]. Sinonasal tract SCC is a rare tumour, and few studies have investigated whether volumetric 18F-FDG uptake parameters predict survival outcomes in the sinonasal tract SCC [6,7,8]. 

We previously reported that a Charlson comorbidity index ≥6 in 79 patients with sinonasal tract SCC predicted shorter both overall survival (OS) and distant metastasis-free survival (DMFS) after various definitive treatments [9] and that gross tumour volume in 24 patients was a predictor of both local recurrence-free survival (LRFS) and disease-free survival (DFS) after chemoradiotherapy [10]. The aim of the present study was to research whether volumetric parameters on F18-FDG-PET/CT are a predictor of survival outcomes in patients with sinonasal tract SCC who undergo various definitive treatments. 

## 2. Materials and Methods

This observation study was retrospectively conducted in accordance with the Declaration of Helsinki. Inclusion criteria of this study was a patient who was both newly diagnosed with sinonasal tract SCC and underwent definitive treatment for resectable disease at the Aichi Cancer Center Hospital from February 2007 to November 2014 [9]. Exclusion criteria of this study was (1) a patient with a serum glucose level ≥200 mg/mL at initial examination and (2) a patient who did not undergo F18-FDG-PET/CT at the Nagoya Radiological Diagnosis Foundation before treatment. We omitted 40 patients with the exclusion criteria from 79 patients with the inclusion criteria and enrolled 39 patients in this study.

### 2.1. Clinical Parameters

Clinical staging was determined based on the seventh edition of the tumour–node–metastasis classification of the International Union Against Cancer. The definitive treatment modality for primary tumour was selected by a multidisciplinary discussion and the patient’s desire for surgery. The other methods of chemotherapy and the Charlson comorbidity index of 19 comorbid conditions were previously described in detail [9].

### 2.2. F18-FDG Uptake Parameters

We assessed a semiquantitative evaluation for the volumetric region of interest (VOI) in the primary tumour after pretreatment with F18-FDG-PET/CT (Biograph True Point PET/CT/40 with True V, Siemens Health Medical Solution Inc., Malven, PA, USA) using the software Advantage Workstation 4.6 programme PET VCAR (GE Healthcare, Chalfont, UK) [5]. The means ± standard deviation (SD) of the blood glucose levels at the initial examination and the durations from F18-FDG-PET/CT to the start of any treatment were 109 ± 19.0 mg/dL and 15.1 ± 12.6 days, respectively. Metabolic tumour volume (MTV) and total lesion glycolysis (TLG) from the VOI were computed by using the threshold fraction of 45% for the maximum of the standardised uptake value (SUVmax).

### 2.3. Statistical Analysis

Associations between F18-FDG uptake parameters (SUVmax, MTV and TLG) were assessed by single linear regression. Associations between the F18-FDG uptake parameters and clinical parameters (age, sex, clinical T and N classification, clinical stage, primary site, treatment group, chemotherapy, Charlson comorbidity index) were compared using the Mann–Whitney *U* test and Spearman’s rank correlation test. Days of survival from the date of F18-FDF-PET/CT to a marked event or last contact were computed using the Kaplan–Meier method. The marked event was death due to sinonasal tract SCC for sinonasal tract SCC-specific survival, death by any cause for overall survival (OS), any recurrence or metastasis for DFS, local recurrence for LRFS, regional recurrence for regional recurrence-free survival (RRFS), and distant metastasis for DMFS. Log-rank test for sinonasal tract SCC-specific survival on monovariable analysis was used to decide the cut-off values for the different F18-FDG uptake parameters. Following the monovariable survival analysis, patients were differentiated into groups based on SUVmax (≥59.0 or <59.0), MTV (≥21.8 or <21.8) and TLG (≥924.6 or <924.6). The monovariate survival outcomes and clinical parameters were compared between the two groups based on MTV (≥21.8 or <21.8) using the log-rank test and Mann–Whitney *U* test, respectively. A Cox regression model with hazard ratio (HR) and 95% confidence interval (CI) were used for the multivariable survival analyses, adjusting MTV (≥21.8/<21.8), age (per 1 year), sex (man/woman), clinical stage (stage IV/stage I–III) and treatment group (radiotherapy/surgery). All statistical analyses were conducted using JMP software, version 9 (SAS, Cary, NC, USA). *p* values less than 0.05 were considered statistically significant.

## 3. Results

**Figure 1:**
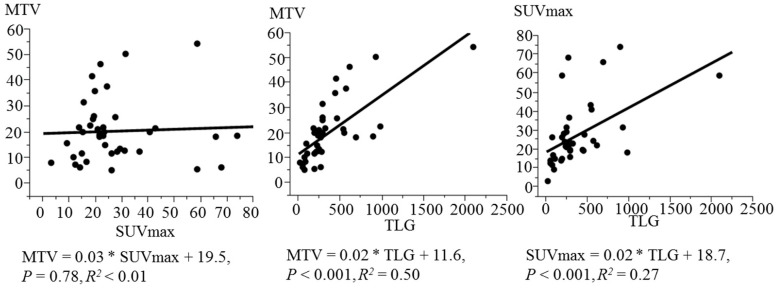
Simple linear regression for F18-FDG uptake parameters (SUVmax, MTV and TLG) in 39 patients with sinonasal tract SCC. F18-FDG, fluorine-18-fluorodeoxyglucose; SUVmax, maximum of standardised uptake value; MTV, metabolic tumour volume; TLG, total lesion glycolysis; SCC, squamous cell carcinoma.

The means ± SD of SUVmax, MTV and TLG of the primary tumours in all 39 patients were 27.4 ± 16.8, 20.4 ± 12.4 and 373.2 ± 373.0, respectively. The association between F18-FDG uptake parameters is shown in Figure 1. TLG was linearly associated with both MTV (*p* < 0.001, *R*^2^ = 0.50) and SUVmax (*p* < 0.001, *R*^2^ = 0.27).

The mean ± SD age at pretreatment was 64 ± 12.9 years (range, 87–28 years) and included 29 men and 10 women. The subsites of the primary tumour were the maxillary sinus (*n* = 28) and nasal and ethmoid sinus (*n* = 11). Table 1 presents the relationship between F18-FDG uptake parameters and clinical parameters. The SUVmax levels (*p* = 0.006) were higher for patients with maxillary sinus than for those with nasal or ethmoid sinus. Clinical T classification was significantly correlated with both MTV (*p* = 0.007) and TLG (*p* < 0.001). Clinical stage was significantly correlated with MTV (*p* = 0.019). 

The means ± SD of follow-up durations were 4.29 ± 3.41 years among all patients, 7.53 ± 1.96 years for 16 patients who remained alive, 2.03 ± 2.11 years for the 23 patients who died and 1.73 ± 1.77 years of the 15 patients who died due to sinonasal tract SCC. Local recurrence, regional recurrence and distant metastasis happened in 13, 5 and 11 patients, respectively.

**Figure 2:**
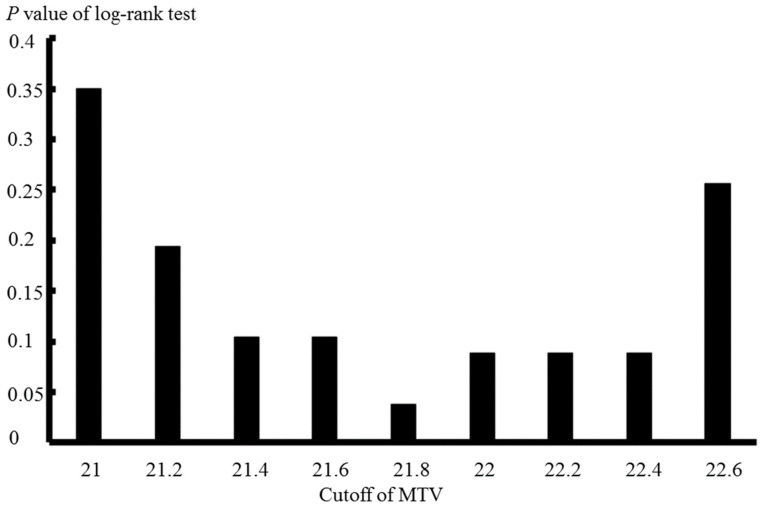
The *p* values of the log-rank test at dissimilar MTV cut-off values in the sinonasal tract SCC-specific survival analysis from 39 patients. MTV, metabolic tumour volume; SCC, squamous cell carcinoma.

Among all patients, the sinonasal tract SCC-specific survival rates at 3, 5 and 10 years were 63.9%, 60.8% and 54.0%, respectively. Based on the log-rank test for sinonasal tract SCC-specific survival, the cut-off values of the lowest *p* value were SUVmax = 59.0 (*p* = 0.18), MTV = 21.8 (*p* = 0.038) and TLG = 924.6 (*p* = 0.05). Association between *p* values of log-rank test and various cut-off values of MTV is presented in Figure 2. 

**Figure 3:**
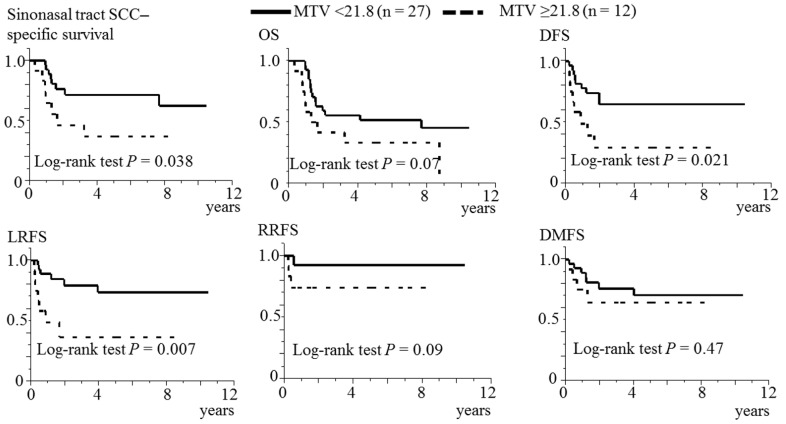
Association between MTV and the survival in 39 patients. MTV, metabolic tumour volume; SCC, squamous cell carcinoma; OS, overall survival; DFS, disease-free survival; local recurrence-free survival; RRFS, regional recurrence-free survival; DMFS, distant metastasis-free survival.

The 5-year OS, DFS, LRFS, RRFS and DMFS rates among all patients were 46.2%, 53.9%, 62.6%, 87.0% and 67.9%, respectively. Figure 3 shows the results of the monovariable survival analyses between MTV ≥ 21.8 and MTV < 21.8. Patients with MTV ≥21.8 had a shorter sinonasal tract SCC-specific survival (*p* = 0.038), DFS (*p* = 0.021) and LRFS (*p* = 0.007) than those with MTV < 21.8. No differences between the two groups of MTV were observed in OS (*p* = 0.07), RRFS (*p* = 0.09) or DMFS (*p* = 0.47).

Table 2 shows no relationships between MTV and clinical parameters.

Table 3 presents the results of the multivariable survival analyses. Age (increase per 1 year) was significantly associated with both longer LRFS (HR = 0.93, 95% CI = 0.88–0.98, *p* = 0.007) and DMFS (HR = 0.94, 95% CI = 0.89–0.99, *p* = 0.018). MTV (≥21.8/<21.8) was significantly related to shorter sinonasal tract SCC–specific survival (HR = 3.69, 95% CI = 1.17–12.0, *p* = 0.026), DFS (HR = 3.38, 95% CI = 1.19–9.71, *p* = 0.023) and LRFS (HR = 5.42, 95% CI = 1.59–20.3, *p* = 0.007).

## 4. Discussion

The present study demonstrated that among 39 patients with sinonasal tract SCC treated with surgery or radiotherapy with or without chemotherapy, an MTV ≥ 21.8 was associated with a shorter sinonasal tract SCC-specific survival, DFS and LRFS in both the monovariable and multivariable analysis after adjusting for age, sex, clinical stage and treatment group.

Several meta-analyses have evaluated volumetric metabolic predictors including MTV and TLG for head and neck SCC as predictors of survival in patients receiving surgical or nonsurgical treatments [2,3]. We have also shown that for patients with SCCs in the hypopharynx (53 patients) who were treated with either surgery or nonsurgery, the MTV is a significant predictor in the univariate OS analysis [5]. The present results demonstrating a significant association between higher MTV and shorter duration of survival are in agreement with these previous results [2,3,5].

Sinonasal tract cancer accounts for approximately 3% of malignant tumours in the head and neck [11]. F18-FDG uptake parameters in the sinonasal tract were evaluated as predictors of variable histological malignancies, including adenocarcinoma and sarcoma [8,12]. For F18-FDG uptake in sinonasal tract SCC, the SUVmax from a single pixel was associated with survival outcomes [7,13], and morphological sphericity predicted local control or failure in 24 patients with chemoradiotherapy [6]. The present results, in which MTV was demonstrated to be a significant predictor, are similar to those of previous reports, which found a significant association between F18-FDG uptake parameters and survival outcomes [6,7,8,12,13].

In terms of the volumetric parameters in sinonasal tract SCC, two previous articles reported that MTV did not predict survival outcomes after chemoradiotherapy [6,7]. A subgroup analysis of 31 patients with only SCC showed that, in 38 patients with various histological malignancies, TLG of the sinonasal tract predicted disease-specific survival after either surgical or nonsurgical treatments, although this article did not assess OS, LRFS, RRFS or DMFS [8]. To the best of our knowledge, the relationship between volumetric F18-FDG uptake parameter and survival outcomes following variable definitive treatments has not been fully investigated. Therefore, we believe this present study contributes to this current need for additional research. Our findings indicate that an MTV ≥ 21.8 in sinonasal tract SCC is associated with a shorter sinonasal tract SCC-specific survival, DFS and LRFS after various definitive treatments by both monovariate and multivariate analysis after adjusting for age, sex, clinical stage and treatment group.

Volumetric F18-FDG uptake parameters were assessed using the thresholds for VOI such as an SUVmax of 2.5–3.0 and 30–50% of SUVmax, and the cut-off value of the F18-FDG uptake parameters was decided by several methods, including lowest *p* value, median value and receiver operating curve analysis [14]. Both 45% of SUVmax for VOI and the lowest *p* value for the cut-off value were used in the same manner for hypopharyngeal SCC [5].

This study has several limitations, including heterogeneous management, a relatively small number of subjects and its retrospective nature. Treatments of primary surgery and radiotherapy in this study were not randomised nor balanced retrospectively. Tumour location and volume from F18-FDG-PET/CT had implicit bias likely to influence the treatment decision making in this study. Because the low sample size and retrospective nature have inherent biases, a future prospective analysis that includes a larger sample size will provide a valid interpretation from significance testing.

## 5. Conclusions

The present study exhibited that a higher MTV is a predictor of survival outcomes (sinonasal tract SCC-specific survival, DFS and LRFS) in patients with only SCC in sinonasal tract cancer who were definitively treated by surgery or nonsurgery.

## Figures and Tables

**Table 1:** Association between F18-FDG uptake parameters (SUVmax, MTV, TLG) and clinical characteristics in 39 patients with sinonasal tract SCC.

Characteristic		*n*	SUVmax	MTV	TLG
Age	Per 1 year	39	27.4 ± 16.8	20.4 ± 12.4	373.2 ± 373.0
	*p* value ^a^		0.90	0.53	0.41
Sex	Man	29	28.0 ± 17.4	21.0 ± 12.8	410.6 ± 420.4
	Woman	10	25.7 ± 15.7	18.9 ± 11.4	264.4 ± 141.2
	*p* value ^b^		0.82	0.72	0.42
	Maxillary sinus	28	31.3 ± 17.6	22.0 ± 12.9	419.0 ± 399.1
Primary site	Nasal or ethmoid sinus	11	17.7 ± 9.3	16.5 ± 10.5	256.5 ± 278.9
Clinical T classification	*p* value ^b^		0.006	0.20	0.10
	cT1	1	11.6	10.3	70.6
	cT2	2	13.2 ± 1.2	6.7 ± 1.0	51.5 ± 2.3
	cT3	9	33.4 ± 18.7	14.7 ± 7.5	259.4 ± 127.1
	cT4a	20	29.4 ± 18.0	23.5 ± 13.4	502.0 ± 473.2
	cT4b	7	20.6 ± 7.70	24.3 ± 12.3	286.6 ± 131.0
Clinical N classification	*p* value ^a^		0.92	0.007	<0.001
	cN0	29	26.7 ± 16.5	18.1 ± 10.5	330.1 ± 272.1
	cN1	3	33.5 ± 22.0	39.2 ± 16.5	960.7 ± 990.7
	cN2	7	27.9 ± 18.2	21.9 ± 12.6	300.0 ± 143.2
Clinical stage	*p* value ^a^		0.81	0.08	0.37
	I	1	11.6	10.3	70.6±
	II	2	13.1 ± 1.2	6.7 ± 1.0	51.5 ± 2.3
	III	8	29.0 ± 14.4	15.8 ± 7.2	258.2 ± 135.8
	IVA	21	31.2 ± 19.5	22.7 ± 13.6	490.9 ± 464.1
	IVB	7	20.6 ± 7.70	24.3 ± 12.3	286.6 ± 131.0
Treatment group	*p* value ^a^		0.84	0.019	0.07
	Surgery	25	25.8 ± 14.1	19.4 ± 13.3	368.5 ± 432.5
	Radiotherapy	14	28.4 ± 18.3	22.3 ± 10.6	381.6 ± 247.5
	*p* value ^b^		0.93	0.25	0.33
Chemotherapy	Presence	31	29.7 ± 17.3	20.2 ± 12.7	395.5 ± 401.7
	Absence	8	18.6 ± 11.8	21.3 ± 11.9	286.7 ± 230.3
	*p* value ^b^		0.06	0.61	0.58
Charlson	<6	37	27.9 ± 17.1	19.8 ± 12.2	354.4 ± 368.5
comorbidity index	≥6	2	18.5 ± 0.6	32.1 ± 13.6	721.2 ± 377.6
	*p* value ^b^		0.28	0.11	0.09

SCC; squamous cell carcinoma, SD; standard deviation, F18-FDG; fluorine-18-fluorodeoxyglucose, SUVmax; maximum of standardized uptake value, MTV; metabolic tumour volume, TLG; total lesion glycolysis. ^a^ Spearman’s correlation or ^b^ Mann–Whitney *U* test was used for statistical analysis.

**Table 2:** Association between MTV and clinical characteristics in 39 patients.

Characteristic		MTV <21.8 (*n* = 27)	MTV ≥21.8 (*n* = 12)	*p* Value
Age	Mean ± SD	65.1 ± 14.5	61.6 ± 8.0	0.27 ^a^
Sex	Man/woman	20/7	9/3	1.00 ^b^
Primary site	Maxillary sinus/others	19/8	9/3	1.00 ^b^
Clinical T classification	cT1-4a/cT4b	24/3	8/4	0.17 ^b^
Clinical N classification	cN0/cN1-2	22/5	7/5	0.23 ^b^
Clinical stage	cStageI–III/cStageIV	10/17	1/11	0.12 ^b^
Treatment group	Surgery/radiotherapy	18/9	7/5	0.72 ^b^
Chemotherapy	Presence/absence	22/5	9/3	0.68 ^b^
Charlson comorbidity index	<6/≥6	27/0	10/2	0.09 ^b^

Abbreviation: SD: standard deviation, MTV: metabolic tumour volume. ^a^ Spearman’s correlation or ^b^ chi-square test was used for statistical analysis.

**Table 3:** Multivariable survival analysis in sinonasal tract SCC by Cox’s proportional hazards model.

Parameter	Sinonasal Tract SCC-Specific Survival	OS	DFS	LRFS	RRFS	DMFS
Age (per 1 year)						
Hazard ratio	0.96	1.00	0.96	0.93	0.99	0.94
95% confidence interval	0.91–1.00	0.97–1.05	0.92–1.01	0.88–0.98	0.91–1.10	0.89–0.99
*p* value	0.07	0.89	0.09	0.007	0.83	0.018
Sex (man/woman)						
Hazard ratio	0.28	0.68	0.41	0.54	0.37	0.63
95% confidence interval	0.08–1.00	0.24–2.13	0.13–1.34	0.14–2.32	0.05–3.32	0.16–3.18
*p* value	0.05	0.49	0.14	0.39	0.35	0.55
Clinical stage (IV/I–III)						
Hazard ratio	1.75	1.16	1.29	0.74	1.07	3.18
95% confidence interval	0.45–8.73	0.38–3.91	0.36–5.19	0.14–3.31	0.09–25.1	0.65–24.0
*p* value	0.43	0.80	0.70	0.69	0.96	0.16
Treatment group (radiotherapy/surgery)						
Hazard ratio	0.64	1.31	0.94	1.16	0.73	0.56
95% confidence interval	0.16–2.42	0.46–3.71	0.27–3.21	0.26–4.96	0.08–6.85	0.11–2.39
*p* value	0.52	0.60	0.93	0.84	0.77	0.44
MTV (≥21.8/<21.8)						
Hazard ratio	3.69	2.25	3.38	5.42	4.74	1.64
95% confidence interval	1.17–12.0	0.89–5.51	1.19–9.71	1.59–20.3	0.70–42.8	0.40–6.24
*p* value	0.026	0.09	0.023	0.007	0.11	0.47

Abbreviation: SCC: squamous cell carcinoma, OS: overall survival, DFS: disease-free survival, LRFS: local recurrence-free survival, RRFS: regional recurrence-free survival, DMFS: distant metastasis-free survival, MTV: metabolic tumour volume.

## Data Availability

The datasets presented in this study are available from the corresponding author by reasonable request.
